# Proton Beam Radiation Therapy for Oropharyngeal Squamous Cell Carcinoma

**DOI:** 10.14338/IJPT-22-00030.1

**Published:** 2023-04-27

**Authors:** William M. Mendenhall, Jonathan J. Beitler, Nabil F. Saba, Ashok R. Shaha, Sandra Nuyts, Primož Strojan, Heleen Bollen, Oded Cohen, Robert Smee, Sweet Ping Ng, Avraham Eisbruch, Wai Tong Ng, Jessica M. Kirwan, Alfio Ferlito

**Affiliations:** 1 Department of Radiation Oncology, University of Florida College of Medicine, Gainesville, FL, USA; 2 Harold Alfonds Center for Cancer Care, Maine General Hospital, Augusta, ME, USA; 3 Department of Hematology and Medical Oncology, Emory University School of Medicine, Atlanta, GA, USA; 4 Department of Head and Neck Surgery and Oncology, Memorial Sloan Kettering Cancer Center, New York, NY, USA; 5 Department of Radiation Oncology, Leuven Cancer Institute, University Hospitals Leuven, KU Leuven - University of Leuven, Leuven, Belgium; 6 Laboratory of Experimental Radiotherapy, Department of Oncology, University of Leuven, Leuven, Belgium; 7 Department of Radiation Oncology, Institute of Oncology, Ljubljana, Slovenia; 8 Department of Otolaryngology - Head and Neck Surgery and Oncology, Soroka Medical Center, Tel Aviv, Affiliated with Ben Gurion University of the Negev, Beer Sheva, Israel; 9 Department of Radiation Oncology, The Prince of Wales Cancer Centre, Sydney, NSW, Australia; 10 Department of Radiation Oncology, Olivia Newton-John Cancer Centre, Austin Health, Melbourne, Australia; 11 Department of Radiation Oncology, University of Michigan Medicine, Ann Arbor, Michigan, USA; 12 Department of Clinical Oncology, Li Ka Shing Faculty of Medicine, The University of Hong Kong, Hong Kong, China; 13 Coordinator of the International Head and Neck Scientific Group, Padua, Italy

**Keywords:** particle therapy, head and neck cancer, oropharynx, cancer outcomes

## Abstract

**Purpose:**

To discuss the role of proton beam therapy (PBT) in the treatment of patients with oropharyngeal squamous cell carcinoma (OPSCC).

**Materials and Methods:**

A review of the pertinent literature.

**Results:**

Proton beam therapy likely results in reduced acute and late toxicity as compared with intensity-modulated radiation therapy (IMRT). The extent of the reduced toxicity, which may be modest, depends on the endpoint and technical factors such as pencil beam versus passive scattered PBT and adaptive replanning. The disease control rates after PBT are likely similar to those after IMRT.

**Conclusion:**

Proton beam therapy is an attractive option to treat patients with OPSCC. Whether it becomes widely available depends on access.

## Introduction

Increasingly, patients presenting with oropharyngeal squamous cell carcinomas (OPSCCs) are otherwise healthy, middle-aged or older, nonsmoking, nondrinking males with mostly human papilloma virus (HPV)-16–positive tumors and enjoy an overall good prognosis [1]. Treatment consists of definitive radiation therapy (RT) alone or combined with concomitant chemotherapy (CRT) or transoral robotic surgery (TORS) and neck dissection, which may be followed by postoperative RT alone or combined with chemotherapy [2, 3]. The acute toxicity secondary to RT and CRT, whether used definitively or following TORS, is often significant and includes mucositis, xerostomia, dysgeusia, and skin desquamation [4, 5]. A significant proportion of patients require a temporary feeding tube insertion, which likely increases the probability of late dysphagia and aspiration, which may necessitate a permanent gastrostomy. There is significant interest in deintensifying treatment for HPV 16–positive patients with a limited, if any, history of smoking by decreasing the RT dose, eliminating chemotherapy in patients with relatively favorable conditions, and/or using more conformal dose distributions [6–13]. Although intensity-modulated RT (IMRT) may be used to produce dose distributions that are more conformal than those produced with older techniques, the acute and late toxicity remain significant [2, 4, 5]. Proton beam therapy (PBT) has gained increasing interest for its potential to produce even more conformal dose distributions with better sparing of surrounding normal tissues and further reduction of toxicity [14–16]. IMRT gained rapid, wide acceptance in the radiation oncology community despite increased complexity and cost. In contrast, acceptance of PBT has been slower, possibly because it is both more expensive and, consequently, less available. Ambiguity about the potential advantages of protons has led to the development and implementation of prospective randomized trials comparing IMRT and intensity-modulated PBT (IMPT) [17, 18]. Models have been proposed to estimate the payor and societal willingness to pay for the increased cost of IMPT [19–22]. The results of these modeling efforts vary with the assumptions used but they generally conclude that the benefit, if any, is most pronounced in younger patients with earlier-stage disease who have a good prognosis and longer life expectancy [23–26].

Herein we review some of the pertinent literature pertaining to the use of IMPT for patients with OPSCC. We will start with efficacy, then review toxicity data followed by dosimetric considerations.

## Materials and Methods

A literature search of all peer-reviewed journal articles published in the English language between 2004 and 2021 pertaining to the use of PBT to treat patients with OPSCC was conducted via the PRISMA [27] 2020 guidelines (**Figure**). Articles containing data related to comparative dosimetry between IMRT and IMPT, modeling to determine the relative value of IMPT in terms of improved quality of life, toxicity, and cancer control outcomes were reviewed.

**Figure.**
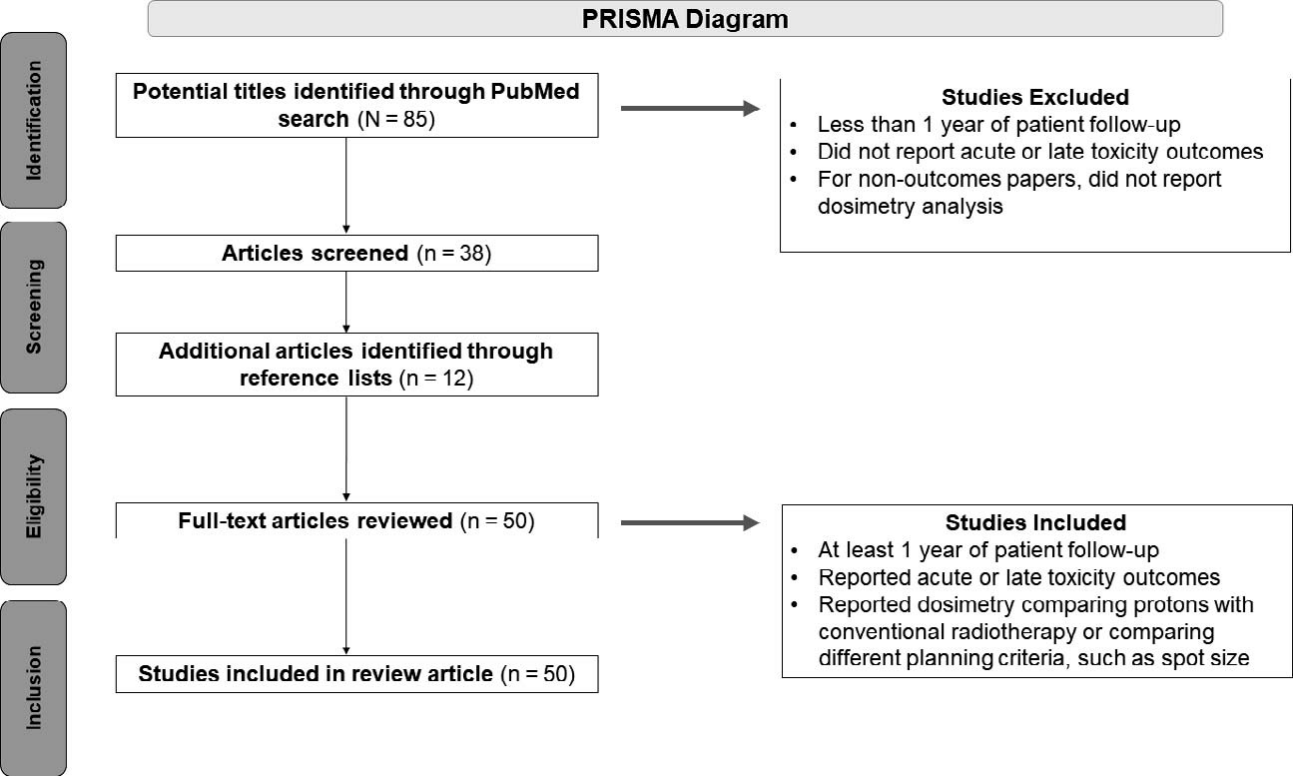
PRISMA diagram illustrating the literature search.

## Results

### Disease Control Outcomes

Slater et al [28] reported one of the first series of patients with OPSCC treated with proton beam RT. A concomitant boost fractionation schedule was used to deliver 75.9 Gy in 45 fractions during 5.5 weeks to 29 patients between 1991 and 2002; follow-up ranged from 2 to 96 months (median, 28 months). The 5-year outcomes were as follows: local control, 88%; regional control, 96%; and local regional control, 84%.

Gunn et al [29] reported on 50 patients with OPSCC treated with IMPT at MD Anderson Cancer Center (MDACC) between 2011 and 2014. Stage III/IV disease was present in 98%: 84% were male, 50% were never smokers, 35% received induction chemotherapy, and 64% received concomitant chemotherapy. Forty-four of 45 tested (98%) were HPV positive. Median follow-up was 29 months. Five patients (10%) had a recurrence. The recurrences were local (1), local and regional (1), regional (2), and distant (1). The 2-year overall survival (OS) was 94.5% and the 2-year progression-free survival (PFS) was 88.6%.

Blanchard et al [8] reported on 150 patients with OPSCC treated with definitive RT at MDACC with either IMRT (100 patients) or IMPT (50 patients) and followed up for a median of 32 months. Some of these patients were likely in the series reported by Gunn et al [29]. Patients were matched 2:1 IMRT to IMPT. There were no significant differences in OS (*P* = .44) or PFS (*P* = .96).

Aljabab et al [16] reported on 46 patients with stage III/IV OPSCC treated at the University of Washington with either definitive IMPT (28 patients; 74-74.4 Gy) or TORS and postoperative IMPT (18 patients; 60-66 Gy). Median follow-up was 19.2 months. Local control was 100%, PFS was 93.5%, and OS was 95.7%. One patient required a salvage neck dissection at 4 months for persistent nodal disease. However, the authors acknowledge limitations of the analysis, such as its retrospective nature, short follow-up period, low event count, and small patient population. Kitpanit et al [30] assessed outcomes in 27 patients treated with definitive (66.7%) or postoperative PBT at the Memorial Sloan Kettering Cancer Center (MSKCC) and followed up for a median of 19 months. At 1 year, the OS, local control, regional control, and distant metastasis–free survival rates were 100%, 100%, 100%, and 96.3%, respectively. One patient had a biopsy-proven lung metastasis 9 months after PBT. Some of the disease control rates after IMPT are depicted in **Table 1**.

**Table 1.** Disease control after proton beam therapy.

**Author and institution**	**No. of patients**	**Median follow-up, mo**	**Local control, %**	**Local regional control, %**	**Disease-free survival, %**	**Overall survival, %**
Blanchard et al [8], MDACC	50	32	NR	91 (3 y)	86.4 (PFS at 3 y)	NR
Slater et al [28], Loma Linda University	29	28	88 (5 y)	84 (5 y)	65 (5 y)	NR
Gunn et al [29], MDACC	50	29	NR	NR	88.6 (2 y)	94.5 (2 y)
Aljabab et al [16], University of Washington	46	19.2	100	NR	93.5 (PFS)	95.7
Kitpanit et al [30], MSKCC	27	19	100 (1 y)	NR	96.3 (1 y)	NR
Yoon et al [31], Samsung Medical Center	67^a^	24.7	NR	NR	82 (PFS at 2 y)	98.4 (2 y)
Manzar et al [32], MDACC and Mayo Clinic	46	12	NR	NR	NR	92.6 (1 y)

**Abbreviations:** MDACC, MD Anderson Cancer Center; NR, not reported; PFS, progression-free survival; MSKCC, Memorial Sloan Kettering Cancer Center; IMRT, intensity-modulated radiation therapy; IMPT, intensity-modulated therapy.

a Combined IMRT and IMPT.

### Toxicity

Sio et al [33] evaluated patient-reported outcomes in a series of patients with OPSCC treated between 2006 and 2015 with definitive RT and chemotherapy, using either IMRT (46 patients) or IMPT (35 patients) at MDACC. Patients completed the MD Anderson Symptom Inventory for Head and Neck Cancer (MDASI-HN) before RT, during RT (acute phase), within 3 months of completing RT (subacute phase), and more than 3 months after RT (chronic phase). The top 5 symptoms on a 0 to 10 scale were as follows: food/taste, mean 4.91; dry mouth, mean 4.49; swallowing/chewing, mean 4.26; lack of appetite, mean 4.08; and fatigue, mean 4.00. Of the top 11 symptoms, there was no difference in symptom burden between IMRT and IMPT during the acute and chronic phases. Of the top 11 symptoms, change in taste and appetite during the subacute and chronic phases favored IMPT. During the subacute phase, the top 5 symptom scores all favored IMPT (*P* =  .013).

Smith et al [34] reported on return-to-work outcomes for 147 patients with OPSCC treated with CRT using either IMRT (78 patients) or IMPT (69 patients). This was a prespecified secondary aim of a prospective phase II/III randomized trial and evaluated absenteeism, pre-absenteeism, and work productivity losses. The percentages of patients working full time in the IMPT and IMRT groups were as follows: pre RT, 60% and 57%; 1 year after RT, 71% and 54%; and 2 years after RT, 78% and 52% (*P* = .06). Higher magnitudes of recovery were noted in patients treated with IMPT for absenteeism (1 year, *P* = .05; 2 years, *P* = .04) and composite work impairment scores (1 year, *P* = .04; 2 years, *P* = .04).

Blanchard et al [8] reported fewer gastrostomy tubes during RT (*P* = .11) and 3 months after RT (*P* = .10) in patients treated with IMPT compared with IMRT. Grade 3 weight loss or placement of a gastrostomy tube 3 months after RT (*P* = .05) and 1 year after RT (*P* = .01) was also reduced in patients treated with IMPT.

Yoon et al [31] reported on 148 patients with OPSCC treated at the Samsung Medical Center between 2016 and 2019 with IMRT (81 patients) or IMRT combined with an IMPT boost (67 patients). A matched pair analysis using propensity score matching of 38 patients from each group was performed. Median follow-up was 24.7 months. For the overall groups, there were fewer cases of grade 3 or higher mucositis (37.0 % versus 13.4 %, *P* < .001) and a lower analgesic quantification algorithm (AQA) score (37.0% versus 19.4%, *P* = .019) for those treated with IMRT/IMPT. For the matched pairs, there were also fewer cases of grade 3 or higher mucositis (39.5% versus 15.8%, *P* = .021) and a lower AQA score (47.4% versus 21.1%, *P* = .016) for the IMRT/IMPT group. Kitpanit et al [30] reported the toxicities in 27 patients with OPSCC treated with either definitive PBT (66.7%) or postoperative PBT (33.3%) at MSKCC, delivered with either uniform scanning (22.2%) or pencil-beam IMPT (77.8%), and followed up for a median 19 months. Among the cohort, the Karnofsky performance status was 90 to 100 and 92.6% were HPV or p-16 positive. Acute grade 3 toxicities were rare (n = 3). One patient had significant dysphagia requiring a temporary feeding tube at 3.4 weeks after PBT initiation, which was removed shortly after treatment. No grade 4 or 5 acute toxicity was observed. Grade 3 late toxicities included hearing impairment requiring a hearing aid (n = 1) and chronic weight loss (n = 2). One patient had dysphagia requiring esophageal dilatation and grade 2 fibrosis. No grade 4 or 5 late toxicities were observed.

Aggarwal et al [35] reported on 906 disease-free OPSCC survivors treated at MDACC with definitive 3-dimensional conformal RT (3D-CRT), IMRT, or proton RT between 2000 and 2013. Median survival at the time of self-reported xerostomia score was 6 years (range, 1-16 years). Moderate to severe xerostomia was reported in 343 respondents (39.1%). Multivariable logistic regression revealed that female sex (*P* = .003), high-school or lower education (*P* = .004), and current smoking (*P* = .016) were predictive of moderate to severe xerostomia. Bilateral IMRT combined with proton RT and ipsilateral IMRT was associated with a lower likelihood of moderate to severe xerostomia than treatment with 3D-CRT. Cao et al [36] reported on 532 patients with OPSCC treated with IMPT (103 patients) or IMRT at MDACC between 2011 and 2015. Patients were asked to complete a xerostomia-specific questionnaire (XQ) every 3 months. Median follow-up was 36.2 months. Moderate to severe xerostomia scores were similar up to 18 months. After 18 months the percentages of patients with moderate to severe xerostomia after IMPT versus IMRT were as follows: 18 to 24 months, 6% versus 20% (*P* = .025); and 24 to 36 months, 6% versus 20% (*P* = .01). During the late phase (24-36 months), high-dose volumes in the oral cavity (V25-V70) were associated with high proportions of patients with moderate to severe xerostomia. In contrast, dosimetric variables for the salivary glands were not associated with late xerostomia.

Grant et al [37] reported on 71 patients with stage III/IV OPSCC treated at MDACC with definitive IMPT who were included in a longitudinal prospective cohort study where swallowing was evaluated at prespecified time points. Tumors were HPV positive in 85.9; 81.4% received bilateral neck RT and 61.8% received concomitant chemotherapy, while 7.0% received induction chemotherapy alone and 14.1% had both concurrent and induction chemotherapy. Swallowing was evaluated with the MD Anderson Dysphagia Index (MDADI). Mean composite MDADI scores were as follows: baseline, 88.2; week 6 of IMPT, 59.6; follow-up at week 10, 74.4; follow-up at 6 months, 77.0; follow-up at 12 months, 80.5; and follow-up at 24 months, 80.1. Poor composite scores (< 60) were as follows: baseline, 5.6%; week 6 of IMPT, 61.2%; follow-up at week 10, 19.1%; 6-month follow-up, 13.0%; 1-year follow-up, 13.5%; and 2-year follow-up, 11.1%.

Manzar et al [32] reported on 305 patients with OPSCC treated at MDACC or the Mayo Clinic with either IMPT (46 patients) or volumetric arc therapy (VMAT) (259 patients) between 2013 and 2018. Patient-reported outcomes were obtained by using European Organisation for Research and Treatment of Cancer Quality of Life, and provider-assessed toxicities were recorded according to the Common Terminology Criteria for Adverse Events (CTCAE), version 4.03 (US National Cancer Institute, Bethesda, Maryland). Median follow-up was 12 months for those treated with IMPT and 30 months for those treated with VMAT. The groups were balanced except that the VMAT patients were older (*P* = .04) and were likely to be smokers (29.3% versus 10.9%, *P* ≤ .01). Patients treated with IMPT required fewer gastrostomy tubes (*P* = .001) and had fewer hospitalizations within 60 days of treatment (*P* < .001). Subgroup analysis showed that the patients who benefited most from IMPT were those treated with definitive RT and CRT. IMPT was associated with reduced end-of-treatment narcotic use and reduced cough and dysgeusia (*P* < .05).

Bahig et al [38] reported on 57 patients with OPSCC treated with IMPT at MDACC between 2011 and 2018. Median age was 60 years, 91% of the cancers were HPV positive, 28% received induction chemotherapy, and 68% received concomitant chemotherapy. Patients were given the Functional Assessment of Cancer Therapy-Head and Neck (FACT-HN) at baseline, during RT, 6 months after RT, 12 months after RT, and 24 months after RT. The percentages of patients completing the FACT-HN were as follows: 6 months, 59%; 12 months, 48%; and 24 months, 42%. FACT-General, FACT-Total, and FACT-Trial Outcome Index score changes were statistically and clinically significant relative to baseline from week 3 of IMPT up to week 2 post IMPT with the nadir at week 6 of IMPT. Maximum scores dropped as follows: FACT-General, 15%; FACT-Total, 20%; and FACT-Trial Outcome Index, 39%. Subdomain scores of physical well-being, functional well-being, and head and neck additional concerns decreased during RT and returned to baseline 4 weeks after completing IMPT.

Zhang et al [39] reported on 584 patients with OPSCC treated with IMRT (534 patients) or IMPT (50 patients) at MDACC between 2011 and 2014. Median follow-up was 33.8 months after IMRT and 34.6 months after IMPT. Mean mandibular doses were as follows: IMRT, 41.2 Gy; and IMPT, 25.6 Gy (*P* < .001). Median time to development of osteoradionecrosis (ORN) was 11.4 months (range, 6.7-16.1 months). The incidence of ORN was 2% after IMPT (1 grade 1) and 7.7% after IMRT (grade 4: 12; grade 3: 5; grade 2: 1; grade 1: 23).

Jain et al [40] reported on 13 patients with HPV-positive OPSCC treated with TORS and postoperative IMPT, treating bilateral fields to 60 Gy in 30 fractions at the University of Pennsylvania. When compared with IMRT, IMPT resulted in significant reductions in the mean doses to the mandible, contralateral parotid, lung, and skin organ equivalent doses (*P* < .001). The authors estimated that the risk of a second malignant neoplasm (SMN) was reduced in all evaluated organs to the extent that IMRT likely results in 4 excess SMNs per 100 patients treated.

Xiang et al [41] used the National Cancer Database, which contains data on 70% of cancer patients treated in the United States, to determine the risk of SMNs in 450 373 patients treated with 3D-CRT (33.5%), IMRT (65.2%), and PBT (1.3%). Included were patients with head and neck, gastrointestinal, gynecologic, lymphomas, non–small cell lung, prostate, breast, bone/soft tissue, and brain/central nervous system cancers. Median follow-up was 5.1 years. There was no difference in the risk of SMNs when comparing 3D-CRT with IMRT (*P* = .75). Significantly fewer SMNs were observed with PBT than with IMRT for all malignancies including head and neck cancer (*P* < .001); however, smoking histories were not available and thus the contribution of this factor to the development of an SMN cannot be established.

The toxicity after IMPT versus IMRT or 3D-CRT is depicted in **Table 2**.

**Table 2.** Toxicity of IMPT compared with IMRT.

**Outcome**	**Reference**
Reduced mucositis	Yoon et al [31]
Reduced analgesic requirements	Yoon et al [31]
Reduced changes in taste and appetite	Sio et al [33]
Fewer gastrostomy tubes	Blanchard et al [8] and Manzar et al [32]
Fewer hospitalizations within 60 d of treatment	Manzar et al [32]
Reduced moderate and severe xerostomia	Smith et al [34]
Reduced mandibular osteoradionecrosis	Zhang et al [39]
Reduced second malignant neoplasms	Jain et al [40] and Xiang et al [41]

**Abbreviations:** IMPT, intensity-modulated proton therapy; IMRT, intensity-modulated photon radiation therapy.

### Dosimetric Considerations

Stützer et al [42] compared the robustness of different planning techniques for pencil beam proton therapy for OPSCC. A cohort of previously treated patients whose plans had been developed without robust optimization—using a single-field optimization technique with planning target volume–based planning to account for uncertainties—was replanned with robust optimization to clinical target volumes (CTVs), using multifield optimization. Multifield optimization resulted in more robust plans with better CTV coverage and organ-at-risk (OAR) dose sparing than single-field optimization planned to the planning target volumes. Wu et al [43] evaluated the impact of adaptive replanning after 4 weeks of IMPT for 10 patients with OPSCC. Mean volumes of all CTVs decreased by 4% to 8% (*P* ≤ .04); volumes of both parotids decreased by 11% to 12% (*P* ≤ .004). All mean doses to CTVs decreased by up to 7% (*P* ≤  .04). Mean doses to the right parotid and oral cavity increased from 5% to 8% (*P* ≤ .03).

Holliday et al [44] reported on 25 patients treated with IMPT for OPSCC between 2011 and 2012 and compared them with 25 patients treated with IMRT between 2000 and 2009. Patients were matched according to whether the fields were unilateral or bilateral, primary site, T stage, N stage, concomitant chemotherapy, induction chemotherapy, smoking status, sex, and age. Mean doses to the anterior and posterior oral cavity, hard palate, larynx, mandible, esophagus, and central nervous system structures associated with nausea and vomiting were all significantly lower for patients treated with IMPT.

van de Water et al [45] reported on 10 patients with OPSCC and a clinically negative neck and compared salivary gland doses resulting from 3D-CRT, IMRT, and IMPT. The authors found that IMPT significantly reduced doses to the OARs, which would likely reduce salivary dysfunction and xerostomia, as compared with 3D-CRT and IMRT. van de Water et al [46] subsequently evaluated reduced spot size in 10 patients with OPSCC treated with IMPT and found that this modality was associated with significantly lower doses to the parotid and submandibular glands.

Deiter et al [47] evaluated factors impacting the necessity for adaptive replanning for 27 patients treated for OPSCC where the bilateral neck was irradiated and found that the dose to the high-dose CTV was primarily impacted by setup variability of the soft tissues of the neck rather than number of beams, dental fillings, and the initial robustness curve of the high-dose CTV.

## Discussion

Proton beam therapy seems to be an excellent treatment option for patients with OPSCC. Although the disease control rates are likely similar to those obtained with IMRT, the first clinical results show a reduction in acute and late toxicity. That said, depending on the endpoint, the improvements are often modest. To limit the use of costly PBT only to patients who are likely to gain the most from proton irradiation, several recommendations have been published and predictive models developed [48]. Currently, the model-based approach, which compares the dose to selected OARs and the resulting difference in Normal Tissue Complication Probability between photon and proton plans, seems to be the most objective approach for patient selection and has also already been tested in clinic [49]. Disadvantages of PBT are lack of availability due to the limited number of facilities and increased cost—which results in an incentive for payors to deny coverage—and lack of mucosal sparing [50, 51]. However, the number of PBT facilities coming online is slowly increasing and there is an incentive for vendors and providers to reduce cost. Additionally, reduced late complications, particularly ORN and hospitalizations and eventual gastrostomies for aspiration, may result in lower health care costs over time. Lastly, quality of life, which is difficult to price, is likely better after PBT because of its ability to reduce xerostomia and swallowing difficulties [19]. Patients with OPSCC are increasingly younger, healthier, and with a longer life expectancy such that reduced late complications and improved quality of life become increasingly important. According to Brodin et al [19, 26], younger nonsmokers/light smokers with p16-posititve tumors may benefit most from PBT, which is cost-effective in more than half of cases involving treatment with comprehensive nodal irradiation. Thus, proton therapy cost-effectiveness varies greatly among patients with OPSCC, which highlights the importance of individualized decision-making. The study of Sher et al [20], who used a Markov model to compare IMRT and PBT, corroborates these findings. The main drawbacks of this study are the limited data available and lower level of evidence due to a lack of randomized clinical comparisons.

There are drawbacks to this review: the data are very limited, the follow-up is short, and most studies involve patients treated at 1 institution so that many of the same patients are included in multiple reports.

## Conclusion

Proton beam therapy is an excellent treatment option for patients with OPSCC. It likely provides disease control rates that are similar to those observed after IMRT but with fewer instances of acute and late toxicity.
